# Supplementary material for: Mortality attributable to seasonal influenza in Greece, 2013 to 2017: variation by type/subtype and age, and a possible harvesting effect
Source: Euro Surveill. 2019 Apr 4;24(14):1800118. doi: 10.2807/1560-7917.ES.2019.24.14.1800118 (PMC6462785; doi:10.2807/1560-7917.ES.2019.24.14.1800118)
Supplement: Supplement S1 [file 1800118_LYTRAS_SupplementS1.pdf]

Supplementary Material for the article "**Mortality attributable to seasonal influenza in Greece, 2013 to 2017: variation by type/subtype and age, and a possible harvesting effect**"

This supplementary material is hosted by *Eurosurveillance* as supporting information alongside the article "Mortality attributable to seasonal influenza in Greece, 2013 to 2017: variation by type/subtype and age, and a possible harvesting effect" on behalf of the authors who remain responsible for the accuracy and appropriateness of the content. The same standards for ethics, copyright, attributions and permissions as for the article apply. *Eurosurveillance* is not responsible for the maintenance of any links or email addresses provided therein.

Supplementary Figure 1: Locations of weather stations recording temperatures.

Supplementary Figure 2: Effect of cubic spline smoothing on type-specific influenza incidence proxies.

Supplementary Figures 3-4: Sensitivity analyses

Supplementary Figure 5: Weekly observed all-cause deaths, deaths attributable to influenza, cold and hot temperatures, and comparisons with (a) model without adjustment for temperature, and (b) model without influenza activity proxies.

Supplementary Figure 6: Subgroup analyses by geographical region

Supplementary Figure 7: Effect of major holidays (Christmas and Easter) on the sentinel surveillance system, and brief description of the ILI rate calculation

Supplementary Figure 8: Number of swab samples tested for influenza in the Greek National Reference Laboratories during the study period, by test result.

Supplementary Figure 9: Lag-response relationships for various temperatures

Supplementary Figure 10: Lag-response relationships for different types of influenza, per age group

Supplementary Figure 11: Exposure-response relationships for different types of influenza, per age group (over all lags)

Supplementary Figure 1: Locations of weather stations recording temperatures.

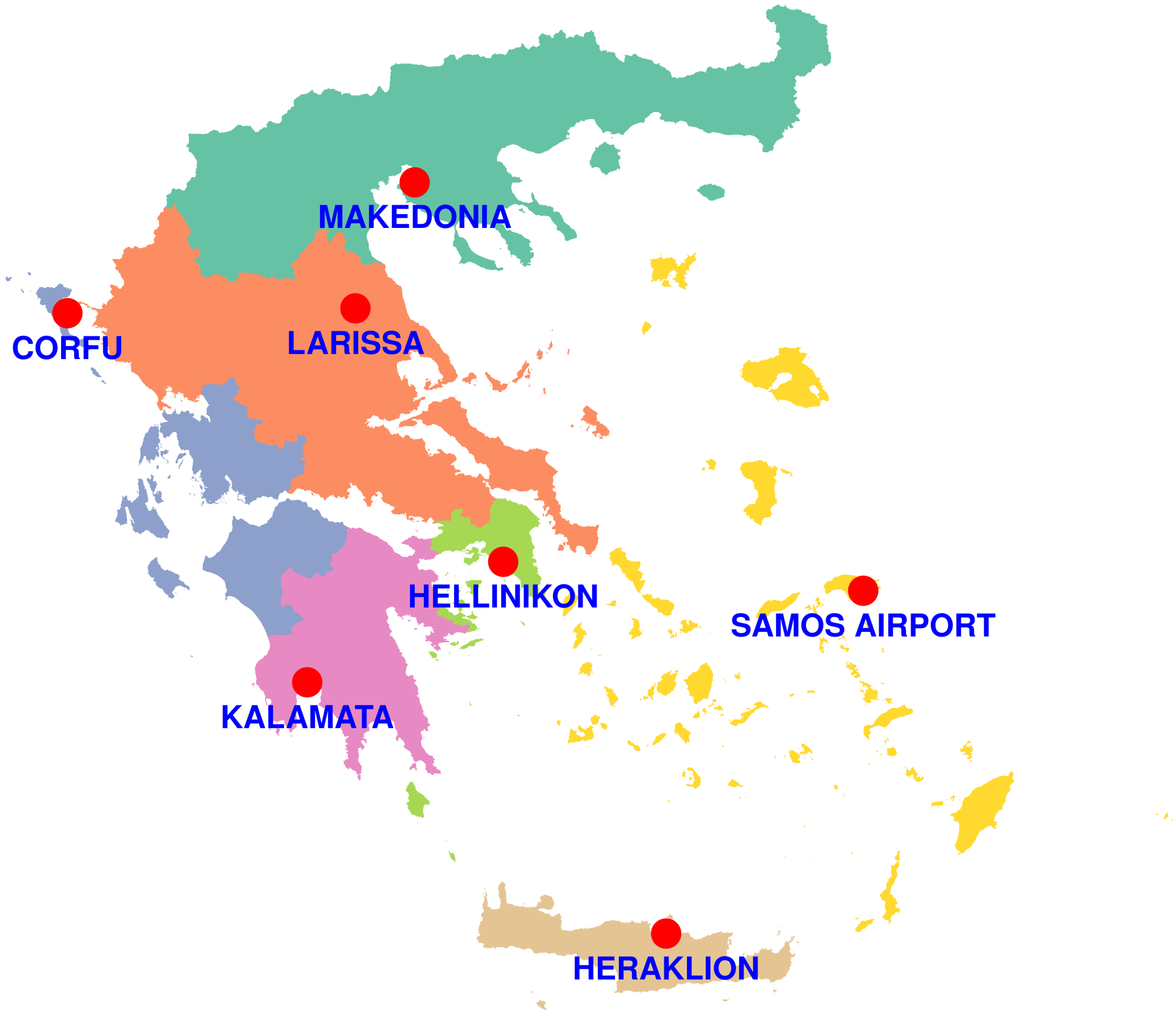

Supplementary Figure 2: Effect of cubic spline smoothing on type-specific influenza incidence proxies.

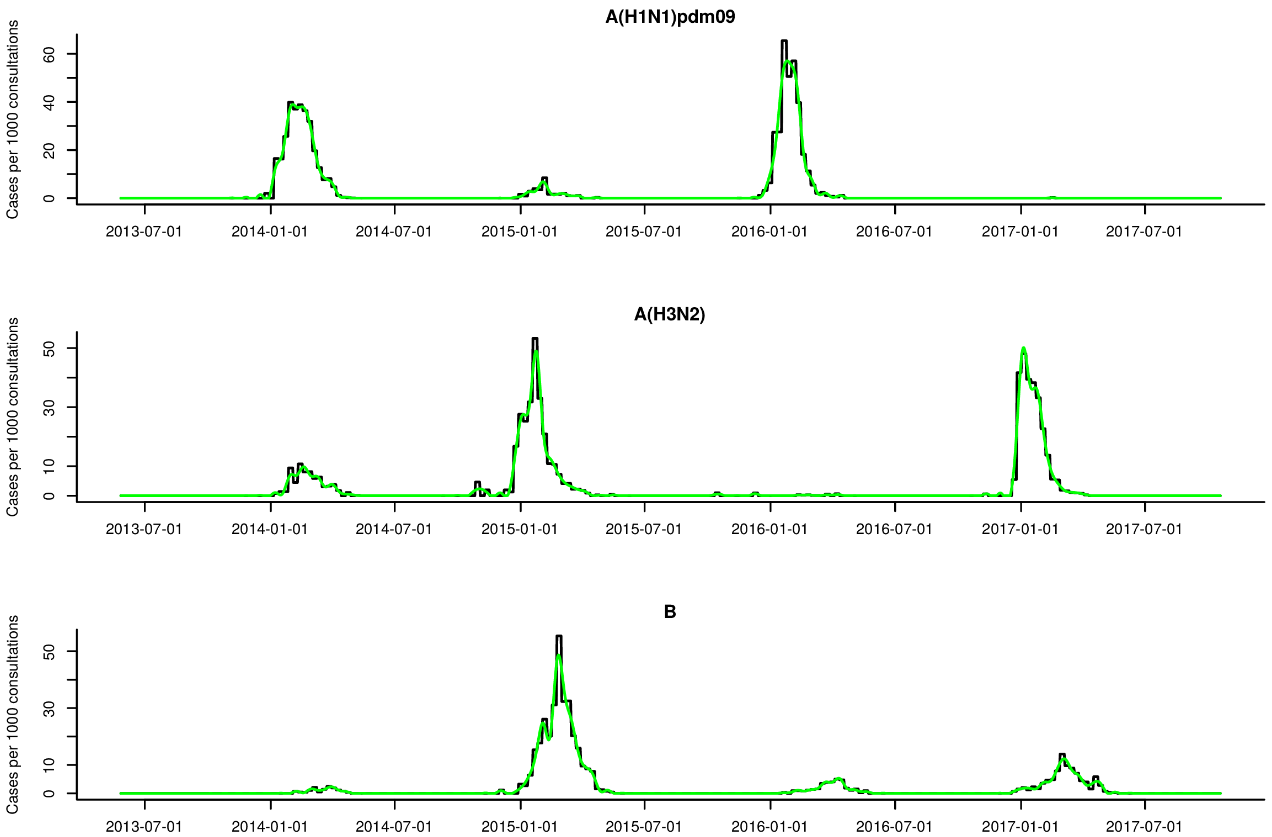

(Black line: unsmoothed, weekly influenza proxy. Green line: smoothed, daily influenza proxy)

SENSITIVITY ANALYSIS: Definition of alternative models

- **Main model:** model for daily mortality, with four cross-basis matrices (average daily temperature, smoothed daily incidence proxies per influenza type), maximum lag 30 days, lag-response relationships as natural cubic spline with 3 knots equidistant at the log scale
- **Model 1a:** as in main model, with a shorter maximum lag of 14 days
- **Model 1b:** as in main model, with a longer maximum lag of 56 days
- **Model 2a:** as in main model, lag-response relationships as natural cubic spline with 3 knots equidistant at the untransformed scale
- **Model 2b:** as in main model, lag-response relationships as natural cubic spline with 9 knots equidistant at the untransformed scale
- **Model 3:** as in main model, without separate incidence proxies per influenza type (two cross-basis matrices, one for temperature and one for all influenza types)
- **Model 4:** as in main model, with no cross-basis matrix for temperature
- **Model 5:** weekly mortality model, four cross-basis matrices (average weekly temperature, weekly incidence proxies per influenza type), maximum lag 4 weeks, stratified lag-response relationships with one indicator variable per week

Supplementary Figure 3: Sensitivity Analysis. Comparison of total number of attributable deaths between alternative models.

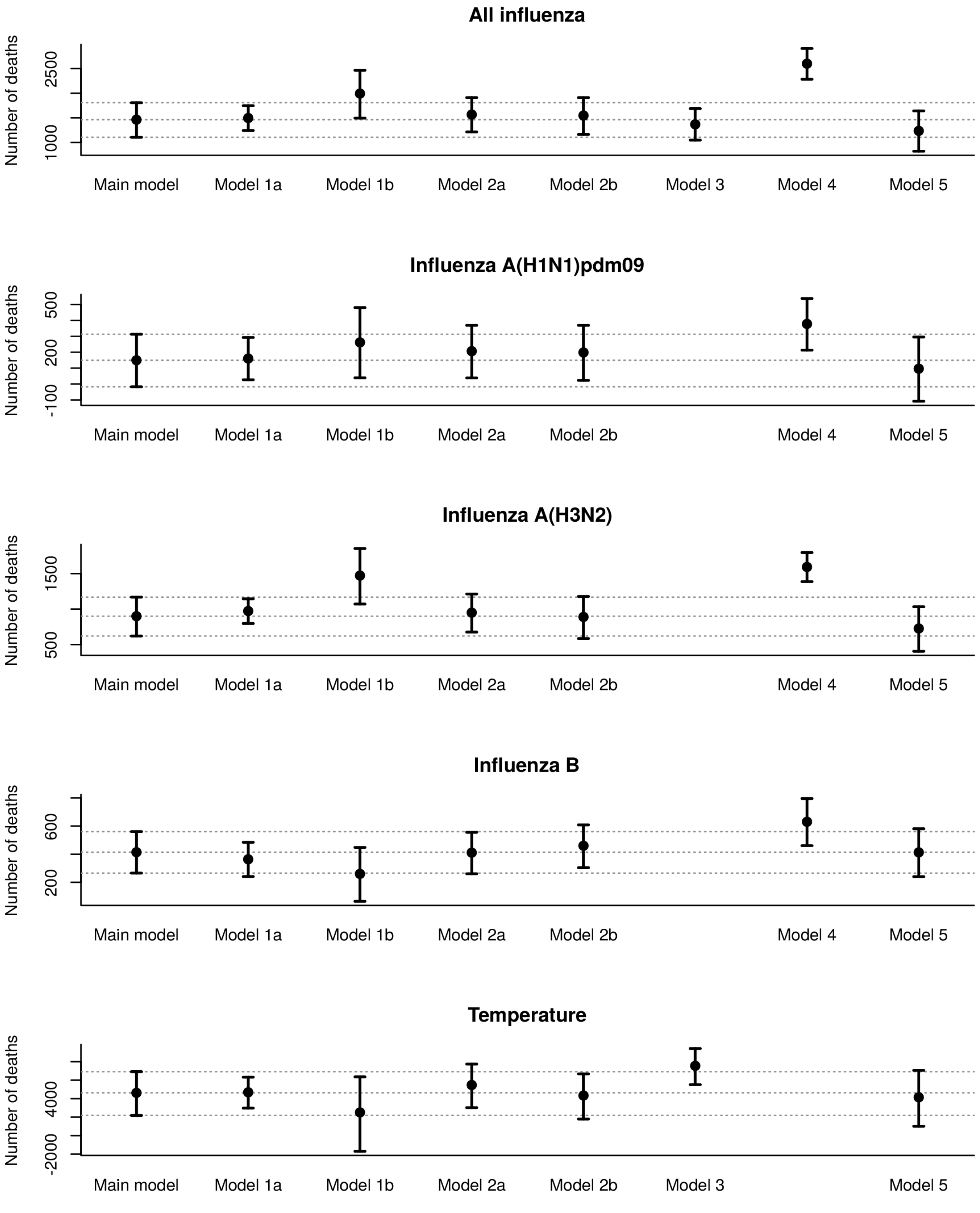

Supplementary Figure 4a: Sensitivity Analysis. Weekly distribution of deaths attributable to all influenza types, comparison between alternative models.

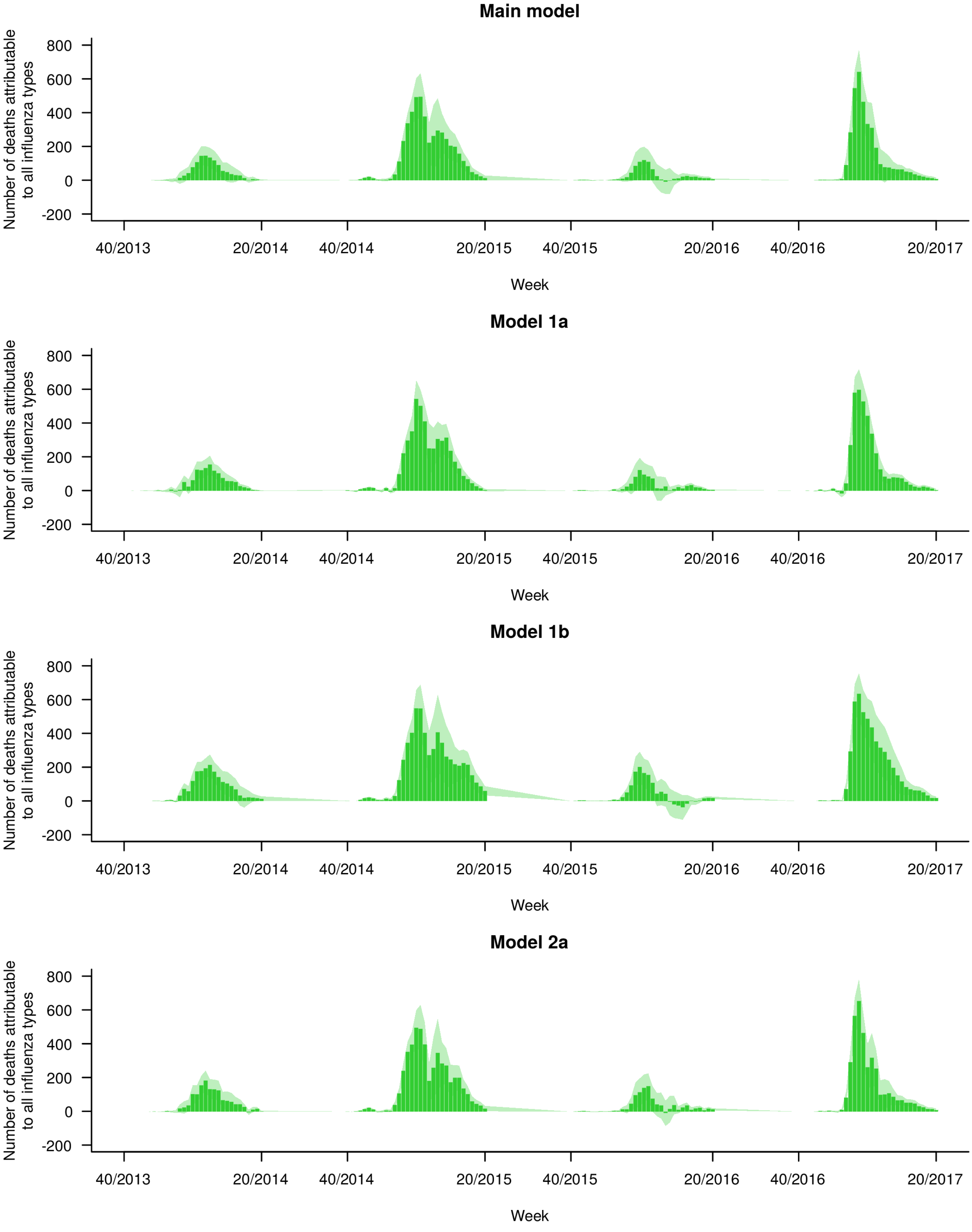

Supplementary Figure 4a (continued)

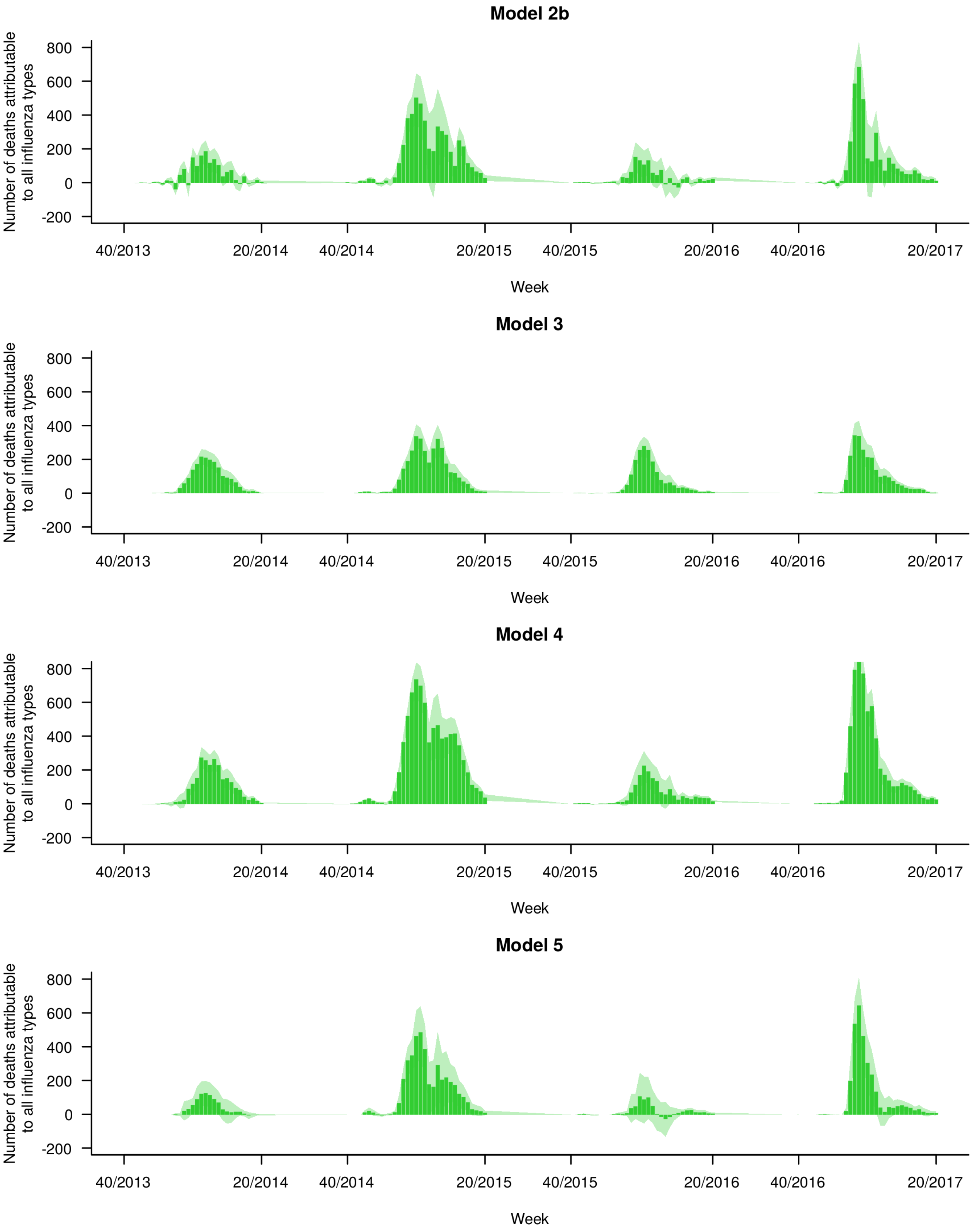

Supplementary Figure 4b: Sensitivity Analysis. Weekly distribution of deaths attributable to influenza A(H1N1)pdm09, comparison between alternative models.

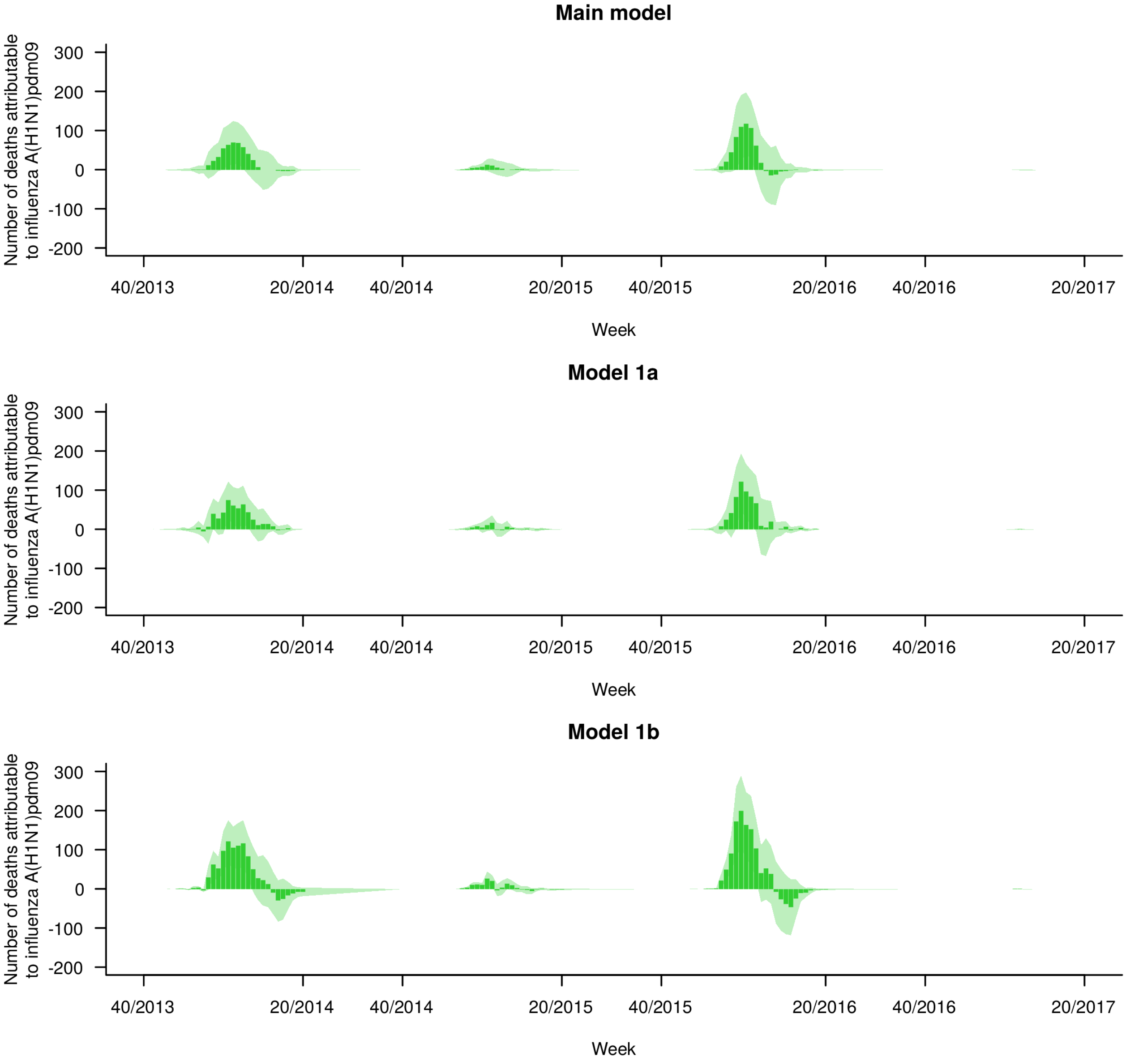

Supplementary Figure 4b (continued)

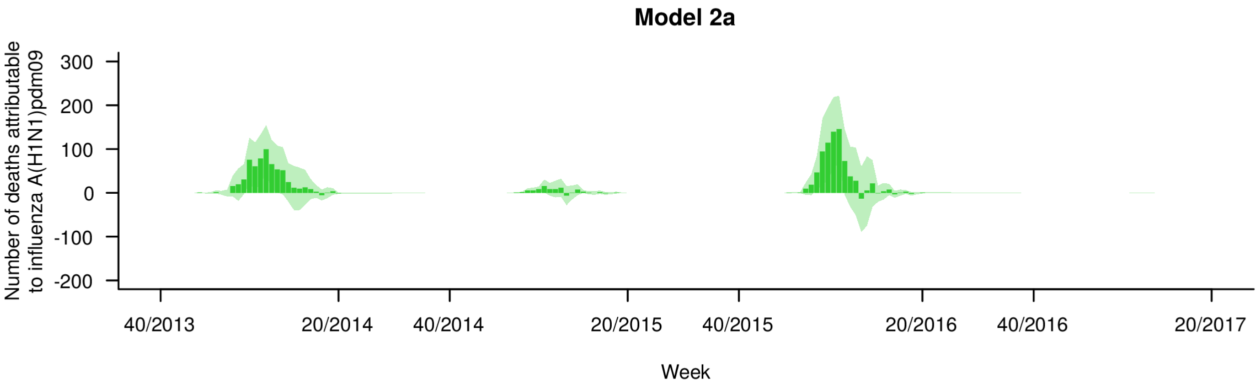

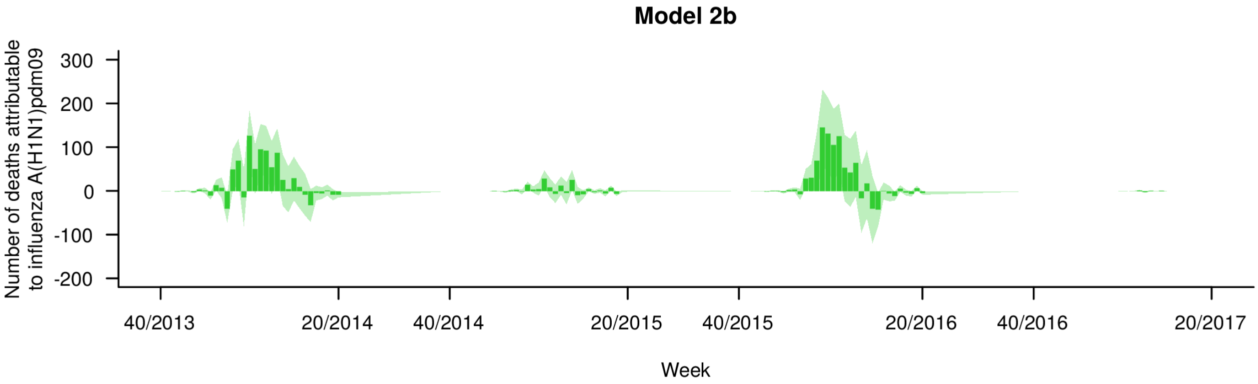

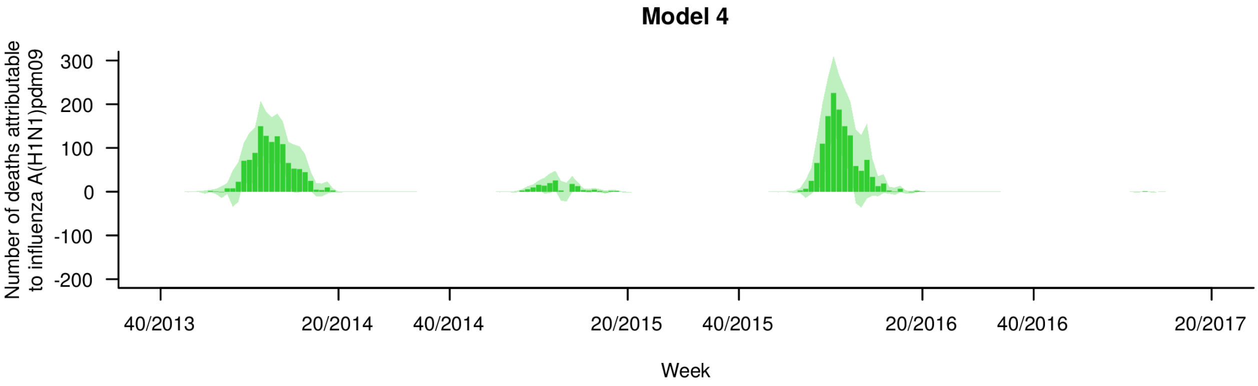

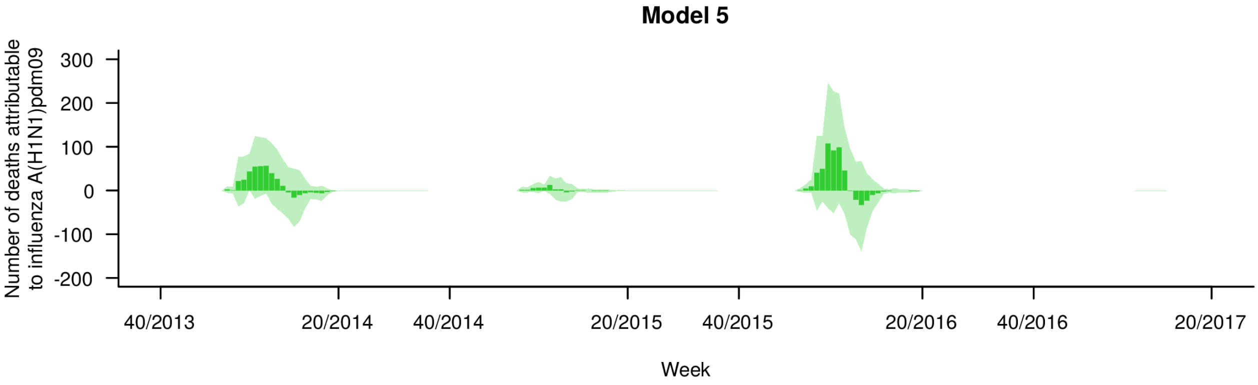

Supplementary Figure 4c: Sensitivity Analysis. Weekly distribution of deaths attributable to influenza A(H3N2), comparison between alternative models.

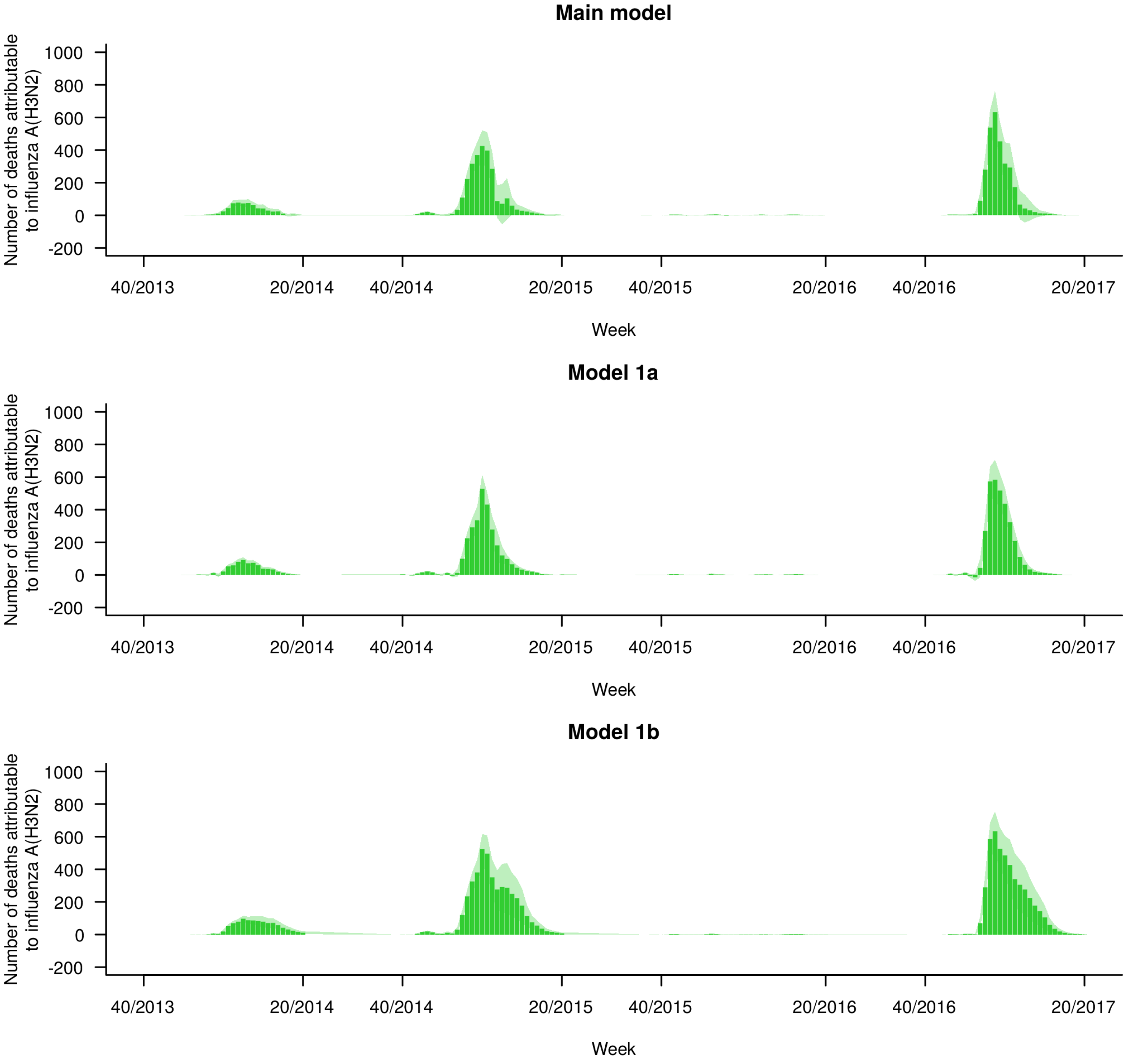

Supplementary Figure 4c (continued)

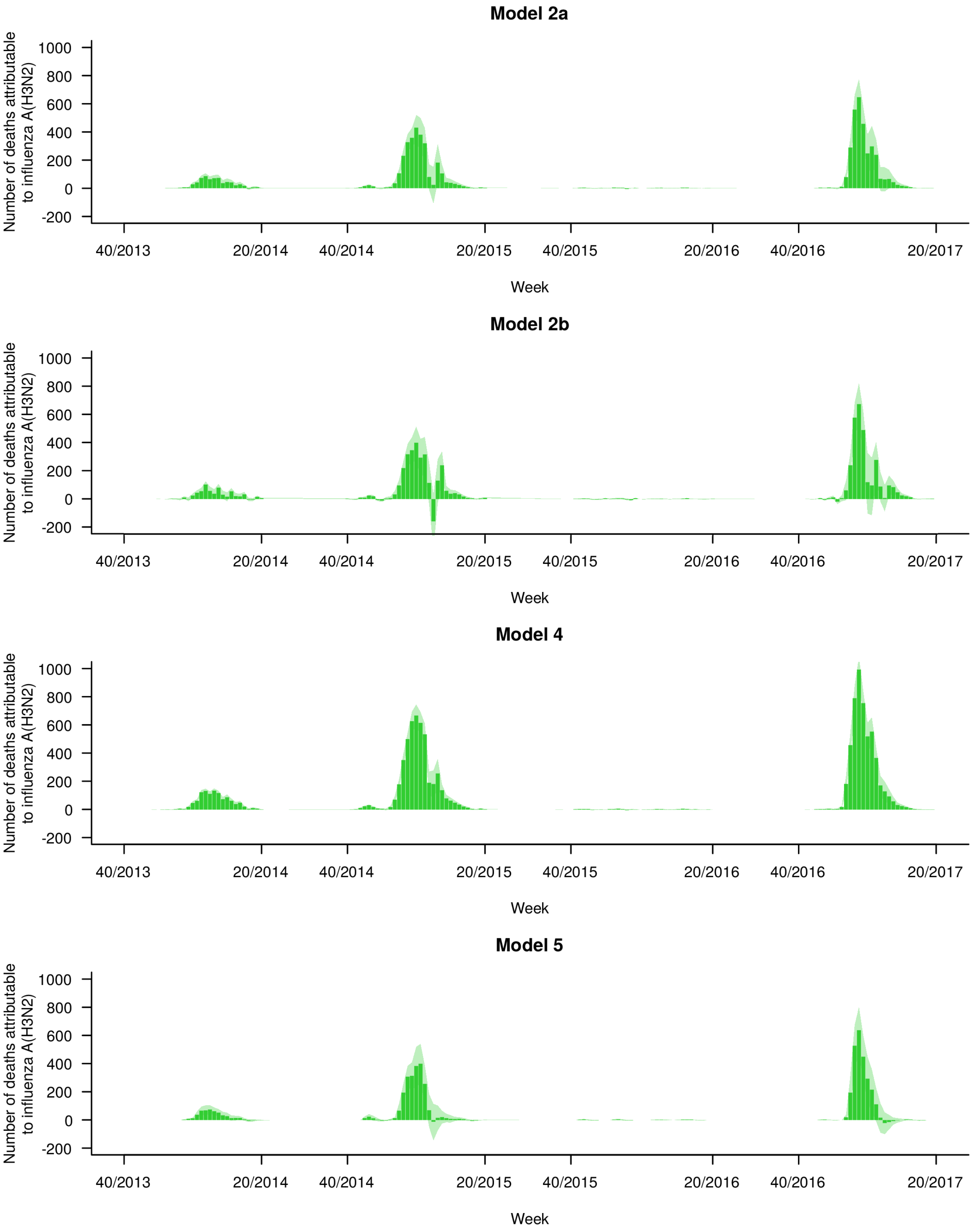

Supplementary Figure 4d: Sensitivity Analysis. Weekly distribution of deaths attributable to influenza type B, comparison between alternative models.

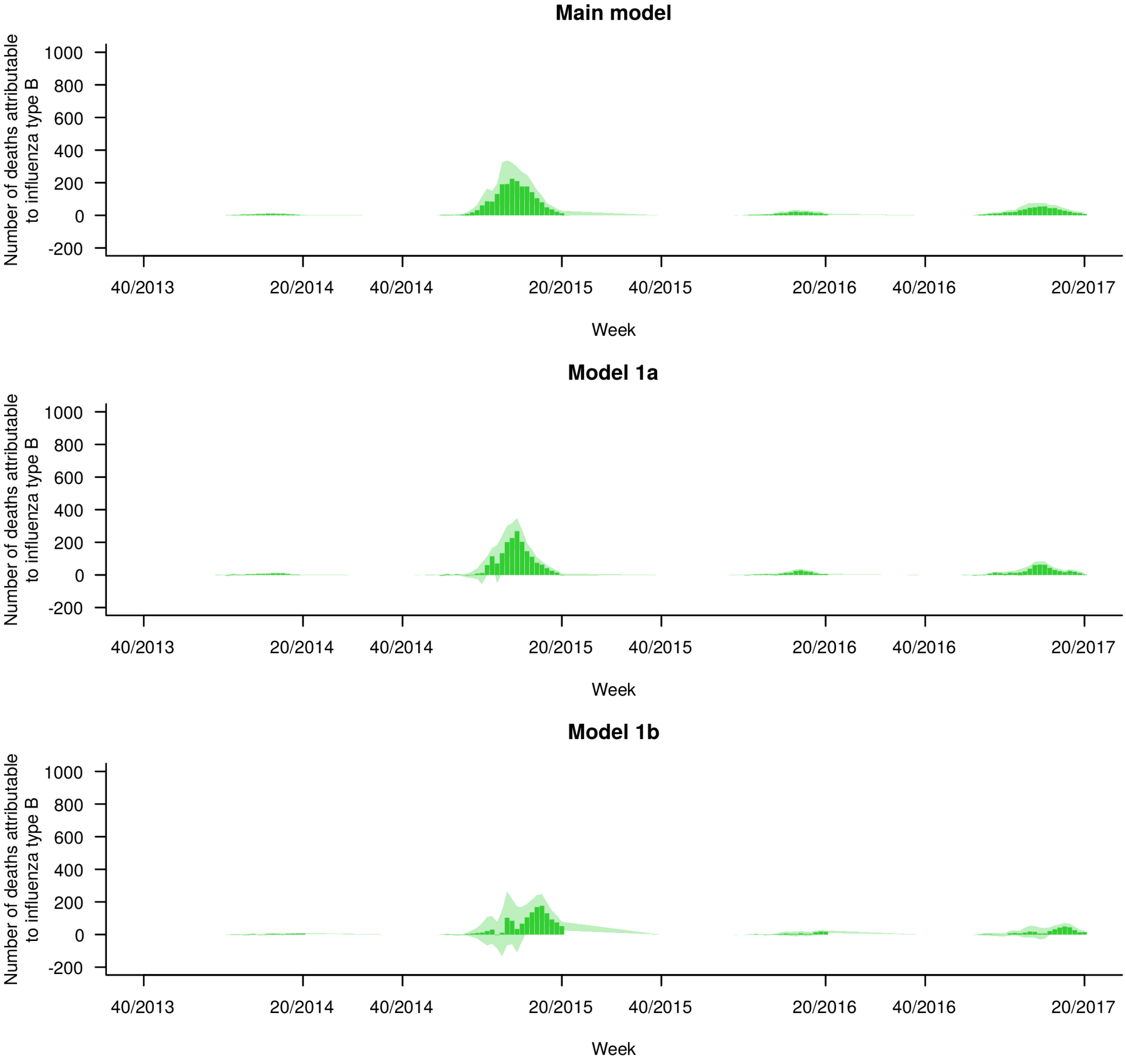

Supplementary Figure 4d (continued)

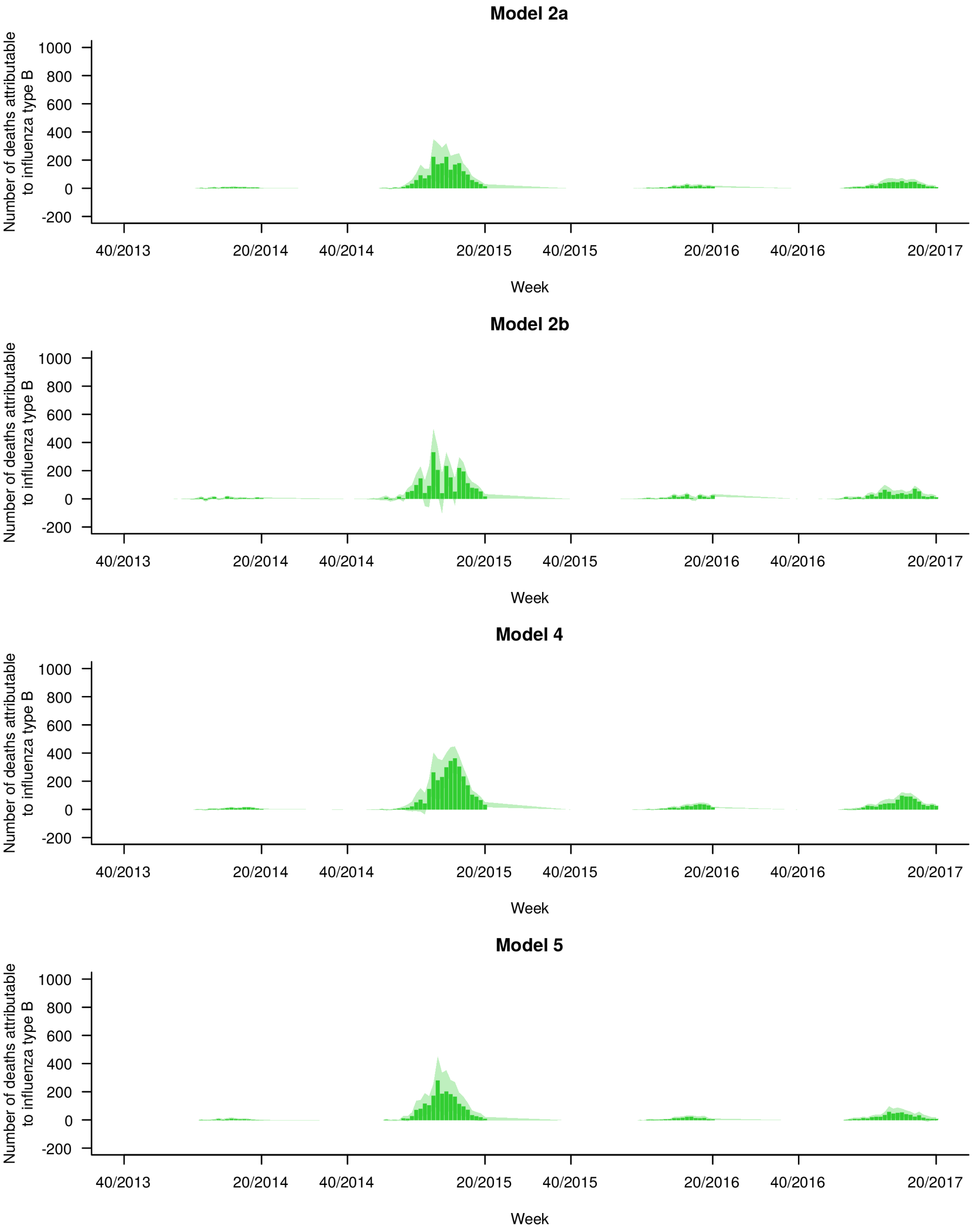

Supplementary Figure 5: Weekly observed all-cause deaths, deaths attributable to influenza, cold and hot temperatures, and comparisons with (a) model without adjustment for temperature, and (b) model without influenza activity proxies, Greece, May 2013 to October 2017.

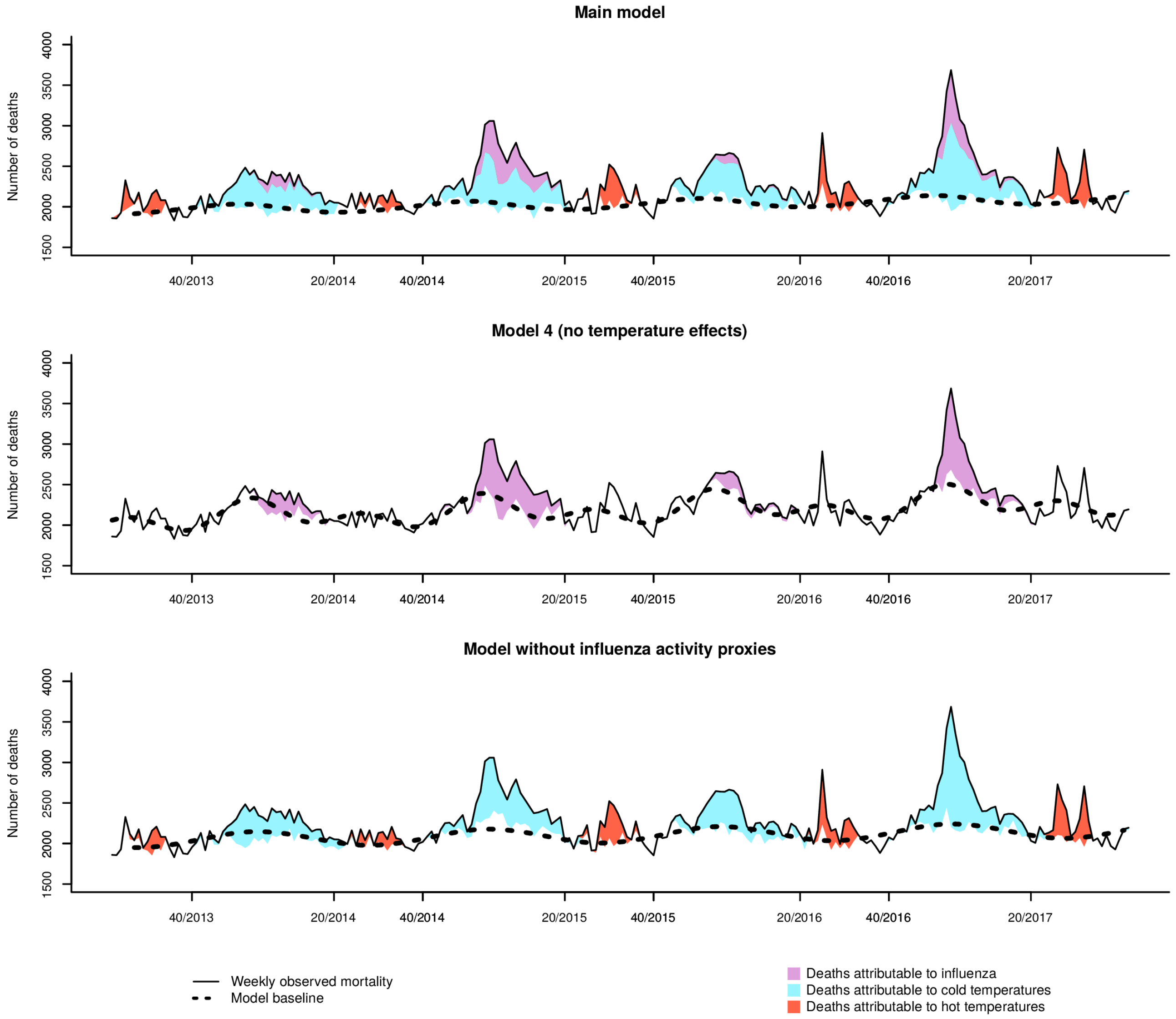

Without adjustment for temperature (sensitivity analysis Model 4), seasonality (in the form of a periodic cubic B-spline term) explains a large part of the variation in observed deaths, and most of the winter excess mortality is attributed to influenza. Conversely, in the main model (which includes a term for temperature in the form of a cross-basis matrix), most of the variation is attributable to non-optimum temperatures and to influenza (to a lesser degree), and seasonality is much less pronounced. Finally, in a model with temperature only and no influenza proxies, nearly all winter excess deaths are attributed to cold temperatures, falsely ignoring the effect of influenza. Both influenza activity and temperature need to be modelled in equal detail, in order to disentangle their effects on mortality.

Supplementary Figure 6: Subgroup analyses by geographical region

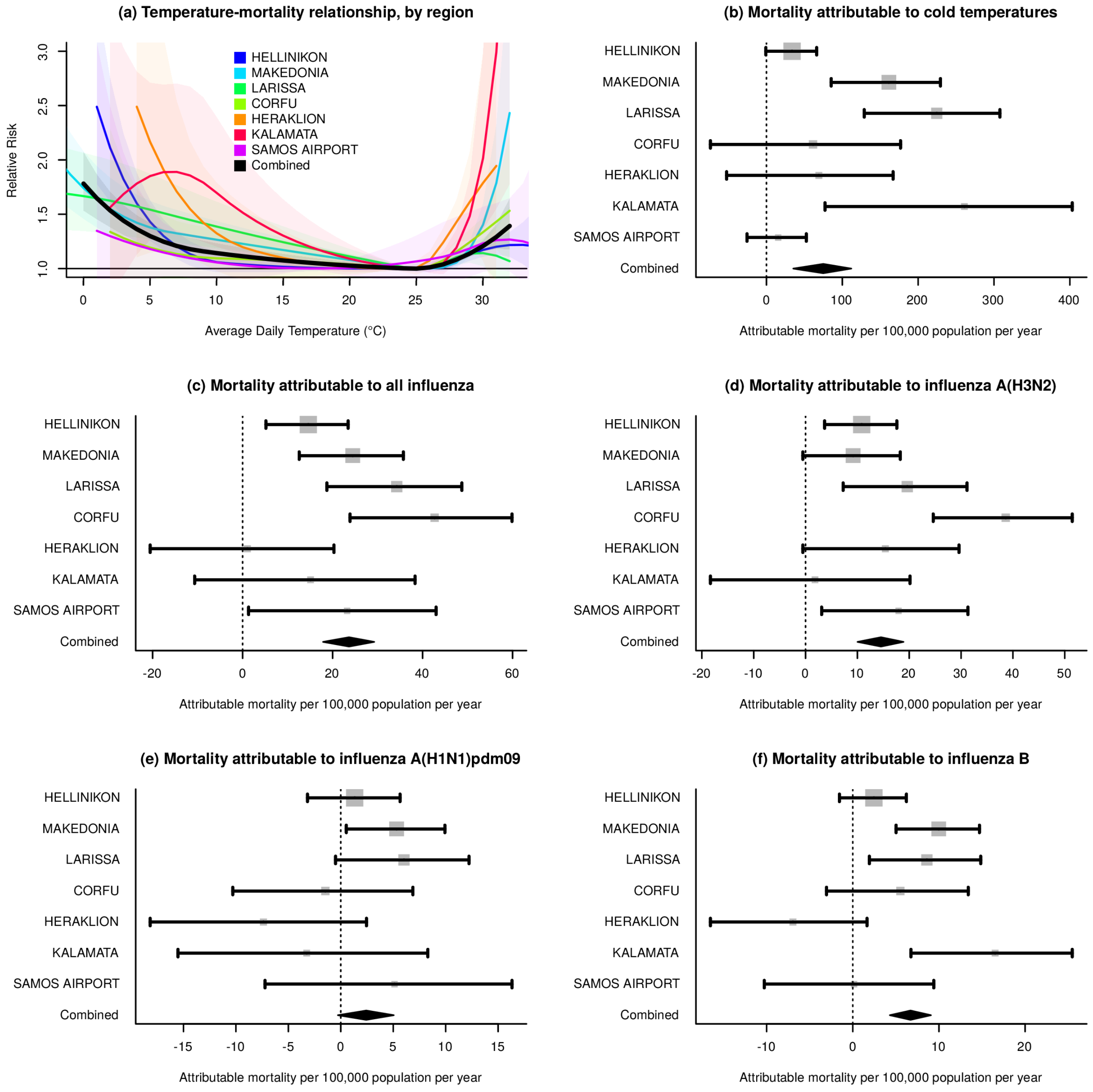

The temperature-mortality association curves are widely overlapping between regions (semi-transparent shaded areas correspond to confidence bands for each curve). Attributable mortality across regions also shows wide overlap (grey square size proportional to the population of each region, and therefore to the region's "weight" on the combined estimate; "combined" refers to the results of the main – countrywide – model, not to a pooled result). Regions are indicated by the name of the weather station, and sorted by population size. Correspondence with administrative regions as follows: Hellinikon (Attica), Makedonia (Macedonia and Thrace), Larissa (Central Greece, Thessaly and Epirus), Corfu (Western Greece and Ionian Islands), Heraklion (Crete), Kalamata (Peloponnese) and Samos airport (North and South Aegean).

Supplementary Figure 7: Effect of major holidays (Christmas and Easter) on the sentinel surveillance system, and brief description of the ILI rate calculation

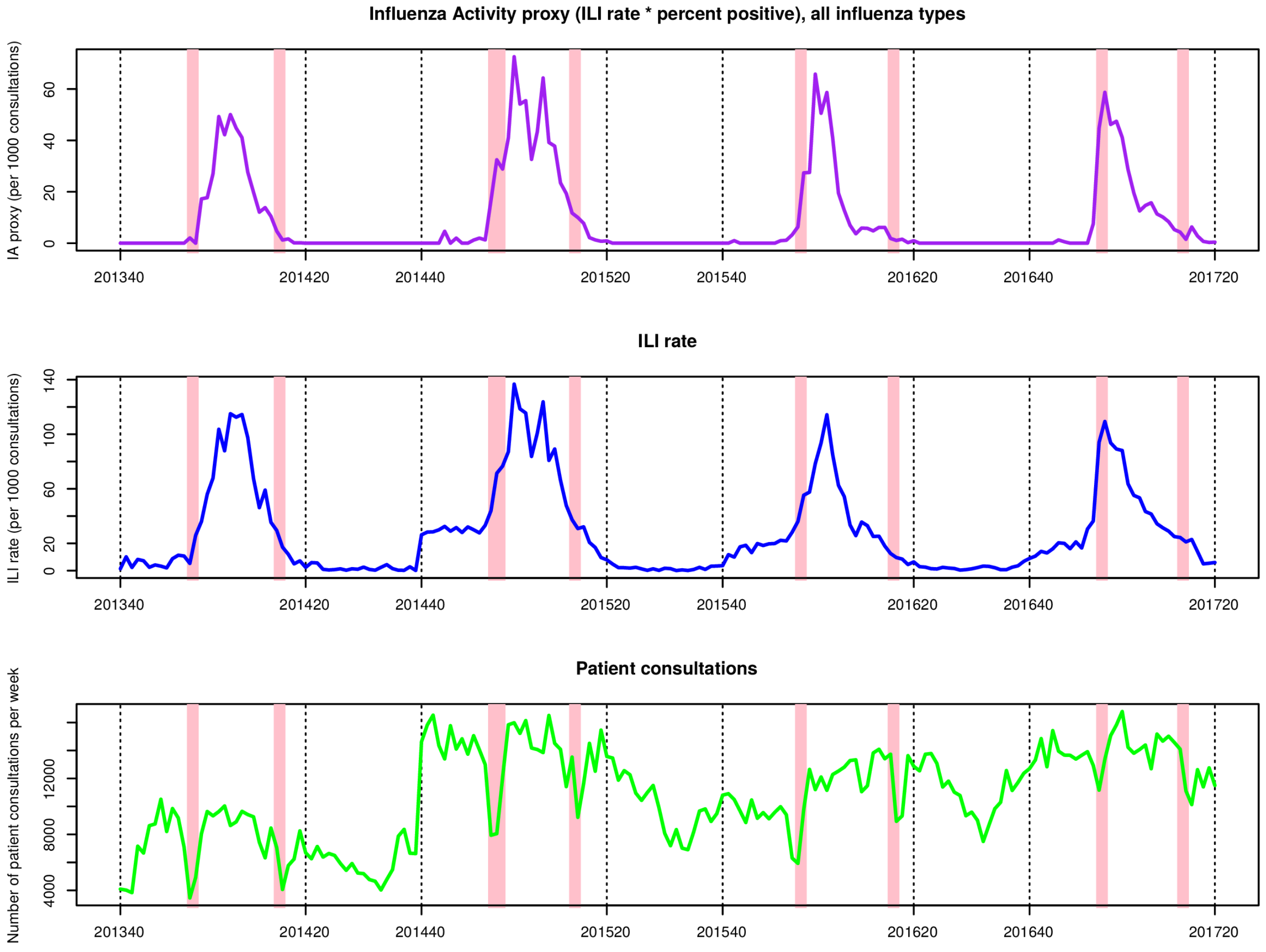

A substantial drop in the number of patient consultations is evident during Christmas and Easter holidays, mostly due to participating sentinel physicians taking their annual leave. However, the effect on the ILI rate and influenza activity proxy (ILI rate times percent positive) remains minimal.

Brief description of the ILI rate calculation

The sentinel surveillance network in Greece, operated by the Hellenic Centre for Disease Control and prevention, consists of a number of primary care physicians selected from across the country with geographic representation. The country is divided into the four NUTS1 regions (Attica, Northern Greece, Central Greece, Aegean islands and Crete), with each further subdivided into rural and urban areas, for a total of eight geographic strata. Each sentinel physician reports on a weekly basis his/her total number of ILI cases (according to the European Union case definition) and total number of patient consultations.

The goal is to estimate the weekly overall ILI rate per 1,000 patient consultations across the country. To do that, a weighted intercept-only random-effects Poisson model is fitted on each week's data: $\log(x_{ij}) = \alpha + Z_j + Z_i + \log(n_{ij})$, where x_{ij} and n_{ij} is the number of ILI cases and total patient consultations, respectively, for sentinel physician i of geographic stratum j. Z_j is a stratum-specific random-effect, and Z_i is an observation-level random effect used to address overdispersion in the data (Harrison XA. PeerJ 2014;2:e616). The exponentiated intercept exp(a) is equal to the ILI rate of the week. To ensure population representativeness, an observation level weight w_j is applied to all observations from stratum j: $w_j = P_j \times \frac{N}{n_j}$, where P_j is the proportion of the population of stratum j to the entire population of the country, n_j is the number of sentinels reporting from stratum j, and N is the total number of sentinels reporting.

This method ensures that the ILI rate is robust to outliers, while simultaneously maintaining population representativeness.

Supplementary Figure 8: Number of swab samples tested for influenza in the Greek National Reference Laboratories, by test result, Greece, influenza surveillance seasons 2013-2014 to 2016-2017.

(a) Influenza surveillance season 2013-2014

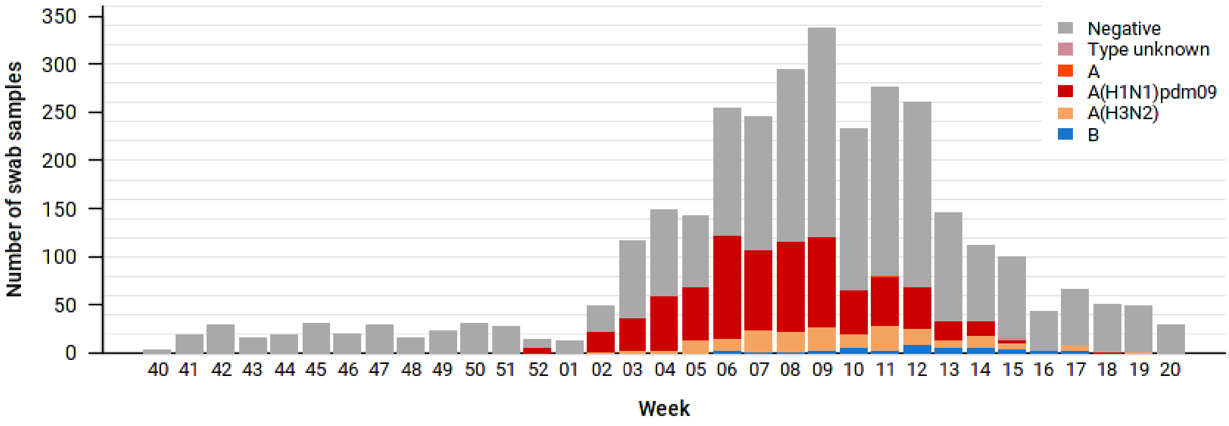

(b) Influenza surveillance season 2014-2015

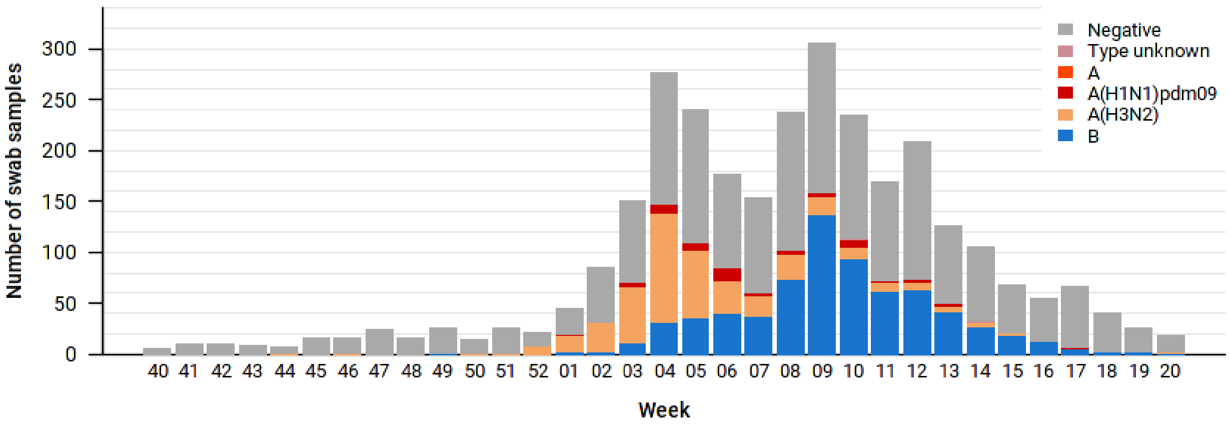

(c) Influenza surveillance season 2015-2016

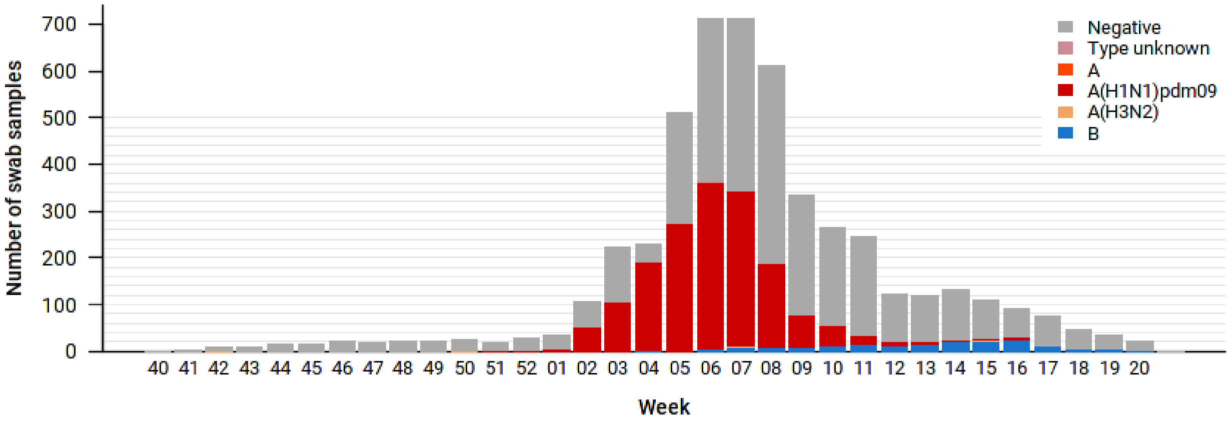

(d) Influenza surveillance season 2015-2016

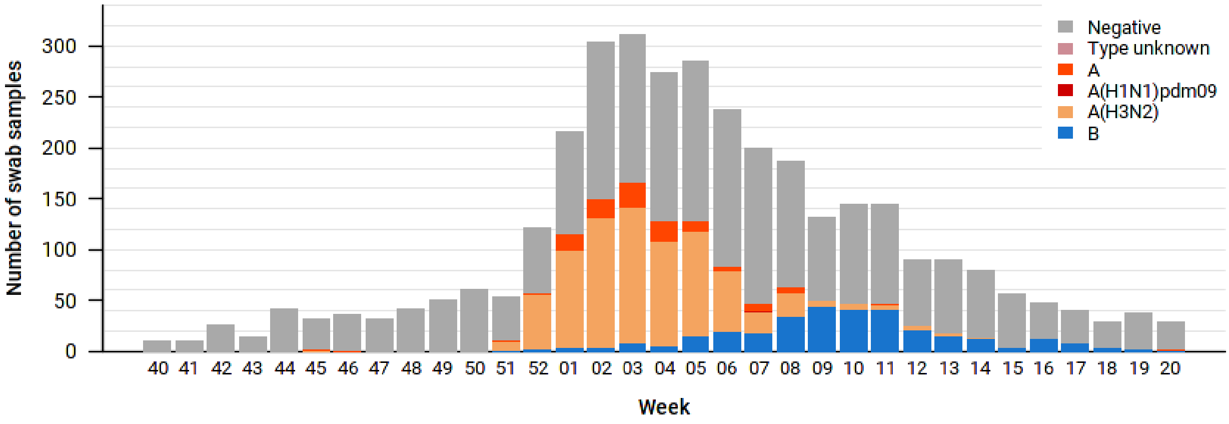

Supplementary Figure 9: Lag-response relationships for various temperatures

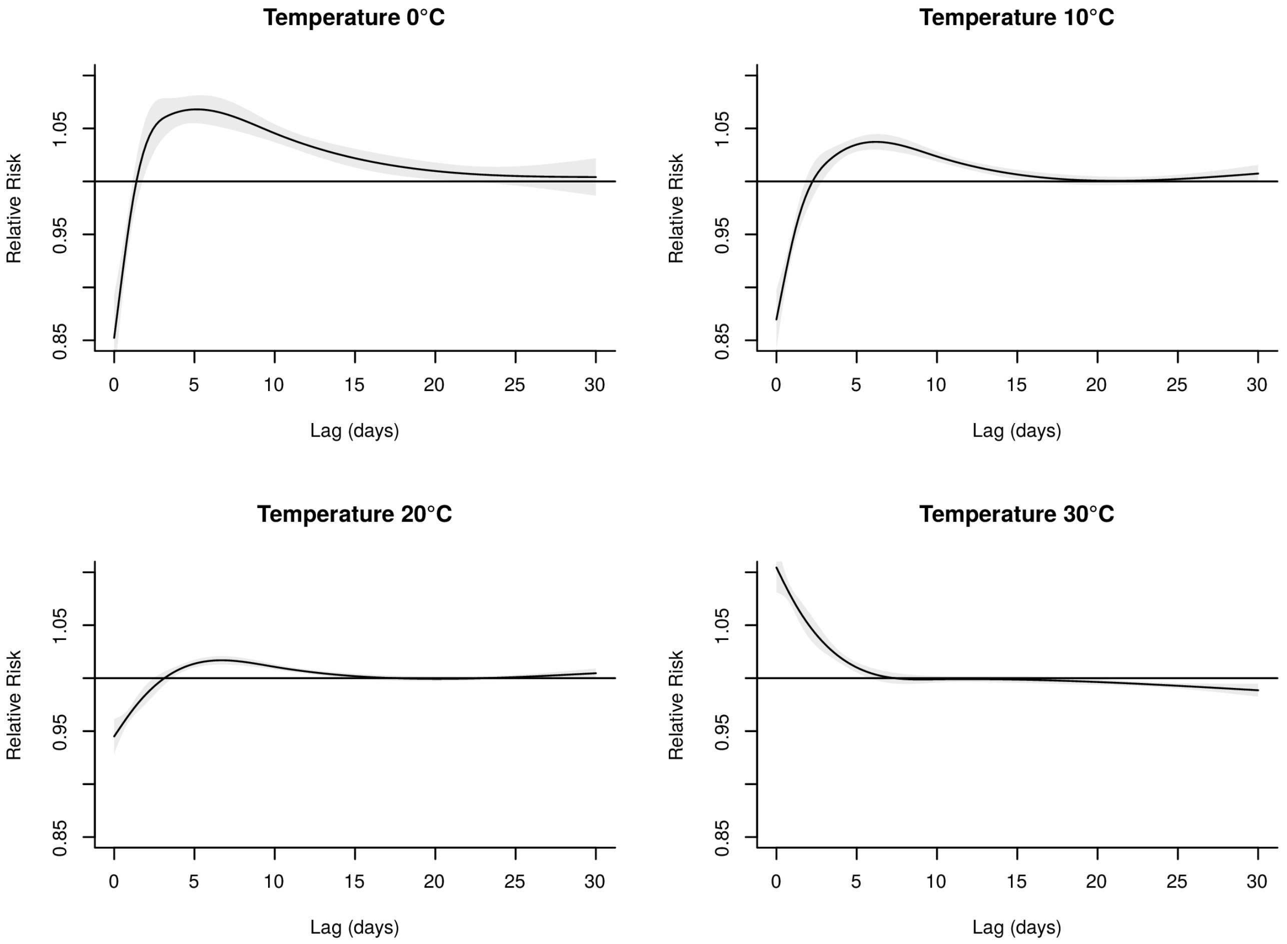

The negative effect of cold temperatures on mortality is more gradual, up to 15 days or more. Conversely, the effect of hot temperatures (30 degrees Celsius) on mortality is much more rapid, within the first few days (up to 7 days).

Supplementary Figure 10: Lag-response relationships for different types of influenza, per age group (for an indicative influenza activity proxy of 30 cases per 1,000 consultations)

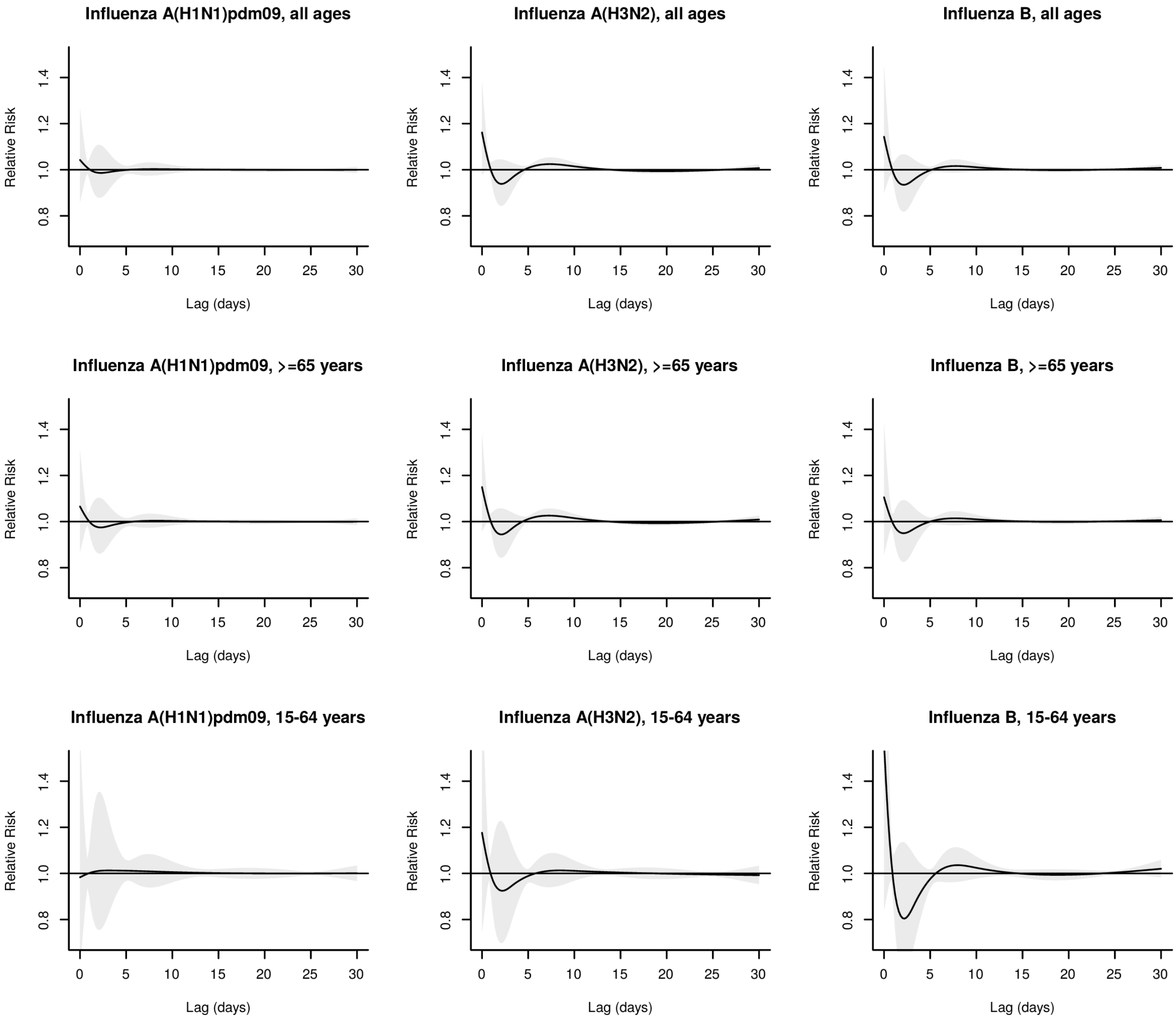

Supplementary Figure 11: Exposure-response relationships for different types of influenza, per age group (over all lags)

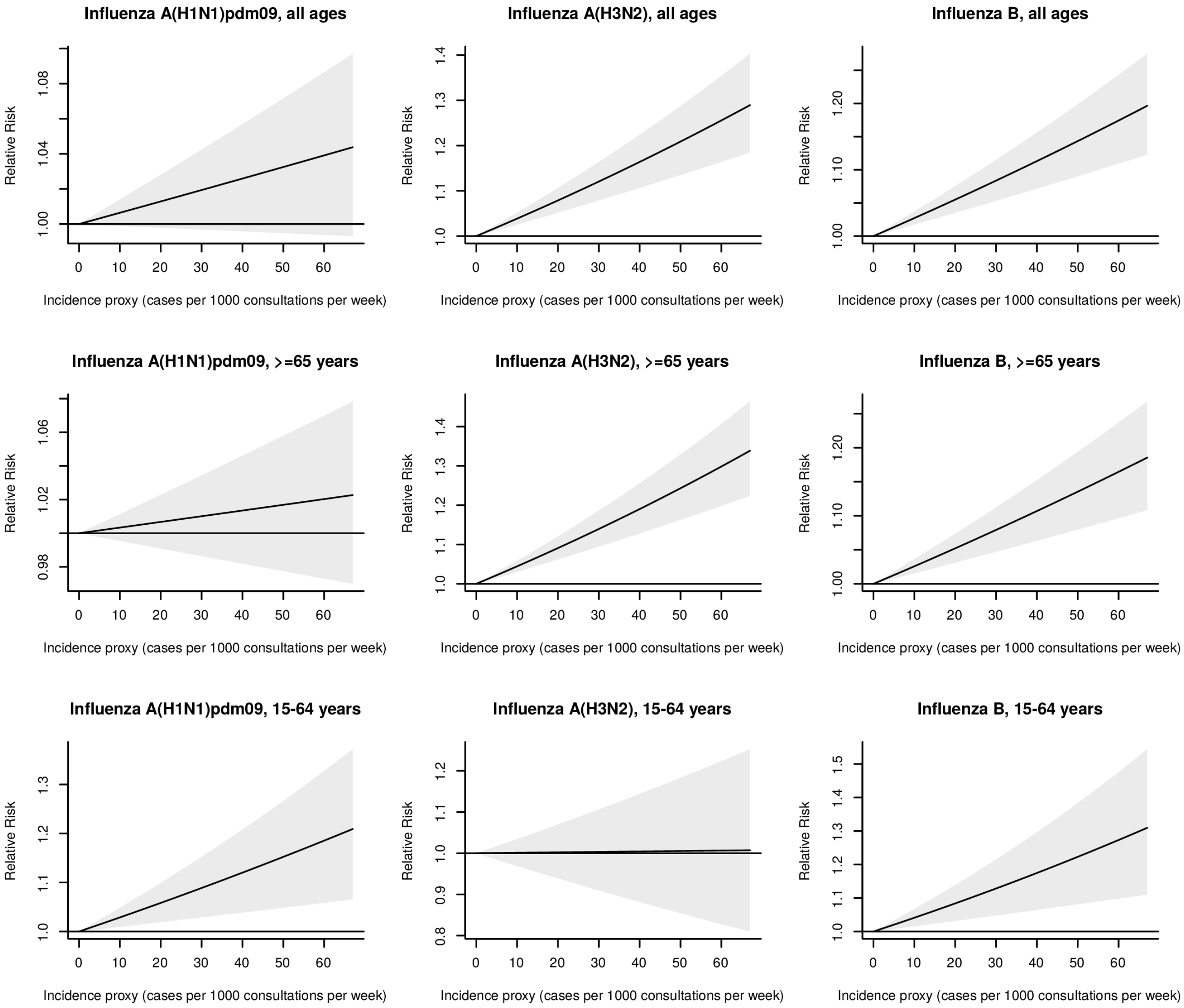

The exposure-response curves are not strictly linear due to the log link used in the Poisson models, but for Relative Risks between 1.0 and 1.5 very closely approximate a linear relationship. This implies a fixed case-fatality ratio per influenza subtype, i.e. a mortality proportional to the incidence of infection with a particular subtype.
